# Influence of Blue Mussel (*Mytilus edulis*) Intake on Disease Activity in Female Patients with Rheumatoid Arthritis: The MIRA Randomized Cross-Over Dietary Intervention

**DOI:** 10.3390/nu10040481

**Published:** 2018-04-13

**Authors:** Helen M. Lindqvist, Inger Gjertsson, Tove Eneljung, Anna Winkvist

**Affiliations:** 1 Department of Internal Medicine and Clinical Nutrition, Institute of Medicine, Sahlgrenska Academy, University of Gothenburg, 405 30 Gothenburg, Sweden; anna.winkvist@nutrition.gu.se; 2 Department of Rheumatology and Inflammation Research, Institute of Medicine, Sahlgrenska Academy, University of Gothenburg, 405 30 Gothenburg, Sweden; inger.gjertsson@rheuma.gu.se (I.G.); Tove.eneljung@vgregion.se (T.E.)

**Keywords:** rheumatoid arthritis, seafood, *Mytilus edulis*, DAS28, patient global health, quality of life (SF-36), diet, intervention

## Abstract

Rheumatoid Arthritis (RA) is a chronic inflammatory disease. This study evaluates the effect of blue mussel intake on disease activity and quality of life in women with RA. Thirty-nine women with established RA and a disease activity score 28 (DAS28) >3.0 were recruited to a randomized 2 × 11-week cross-over dietary intervention. The participants continued with their medication and habitual diet and exchanged one cooked meal a day, five days a week, with a meal including 75 g blue mussels or 75 g meat. Diets were switched after an eight week washout period. Data regarding quality of life (SF-36), blood lipids, erythrocyte sediment rate (ESR), C-reactive protein (CRP) and tender and swollen joints were examined at the start and end of each dietary period. Thirty women completed one period, and twenty-three completed both. Intake of the blue mussel diet led to a significant reduction of DAS28-CRP (*p* = 0.048), but not DAS28. The number of EULAR (European League Against Rheumatism) criteria moderate and good responders were higher when consuming blue mussel diet (*p* = 0.036). Blood lipids did not change. To conclude, blue mussel intake reduced disease symptoms in women with RA and improved perceived health. The reported effects need to be confirmed by non-patient reported outcomes, such as inflammation markers.

## 1. Introduction

Rheumatoid arthritis (RA) is a chronic inflammatory disease, characterized by systemic inflammation and joint damage, that affects 0.5–1% of the population globally, predominantly women [1]. Treatment of rheumatoid arthritis (RA) aims at long-term remission, i.e., absence of: joint tenderness, swelling and destruction, pain and functional impairment. Even though anti-rheumatic therapies have improved tremendously during the last few decades and disease activity is reduced in most patients, pain, disability and/or fatigue are in too many cases persistent [2,3,4]. Pain and fatigue are associated with each other, as well as with depression [5] and are significant predictors for high health care costs, loss of employment and physical functioning and poor quality of life [4]. Lifestyle interventions such as physical activity provide evidence of benefit in relation to self-reported fatigue and pain in adults with RA [6]. However, the effectiveness of dietary changes on inflammation, pain, fatigue and quality of life has not been satisfactorily evaluated as a result of the few well-controlled dietary interventions [6,7]. It is well known that diet and lifestyle are strongly associated with other chronic diseases, such as cardiovascular diseases, cancer and diabetes [8]. Unfortunately, efforts to help patients with RA to improve their diet are hampered by the ambiguous evidence base for the relation between diet and RA. Thus, to optimize not only pharmacological treatment, but also treatment related to lifestyle factors, it is urgent to establish how different foods and diets influence disease activity, i.e., inflammation, swollen and tender joints, pain and general health in patients with RA.

Many nutrients and dietary components are involved in or related to the human immune system and inflammatory processes [9]. Selenium is known to be immune modulating and anti-inflammatory, and although it is thought that glutathione peroxidase plays a role in RA, no convincing evidence exists that selenium supplementation in individuals with adequate status is beneficial [10,11]. The evidence that n-3 PUFA has a modest beneficial effect on symptoms such as morning stiffness, patient assessed pain, fewer tender joints and decreased intake of NSAIDs (Non-steroidal Anti-Inflammatory Drugs) in patients with RA is fairly consistent [12,13]. Fish oil or dietary n-3 PUFA, i.e., reflecting fish intake, in addition to treatment with disease-modifying antirheumatic drugs (DMARD) have also been reported to increase the number of successful and continued DMARD treatments, indicating that pharmacological and dietary treatment complement each other [14,15]. Adam et al. reported that the anti-inflammatory effect (reduced CRP) from fish oil in patients with RA increased when combined with a lacto-vegetarian diet low in arachidonic acid [16]. Thus, exchanging meat for fish or shellfish, which will result in a semi-vegetarian diet rich in marine n-3 PUFA, could be more effective than simply adding fish oil to a mixed diet.

Blue mussels, like fish, contain the marine n-3 PUFA eicosapentaenoic acid (EPA) and docosahexaenoic acid (DHA). In addition, they are rich in nutrients such as zinc, selenium, riboflavin and carotenoids. Fish and shellfish are often regarded as a homogenous food group, even though the content of nutrients varies significantly. Therefore, the effects from different fish or shellfish species cannot be separated in most studies. Intervention studies, in patients or healthy subjects, with blue mussel or shellfish intake have so far not been published. However, there are a few studies on anti-arthritic or anti-inflammatory effects from lipid extracts from mussels: One study on lipid extracts from blue mussel (*Mytilus edulis*) in comparison to olive oil showed positive effects on arthritis in rat [17]. Another recent study on lipid extracts from hard-shelled mussel (*Mytilus coruscus*) in patients with RA showed a reduced disease activity and improvements for some cytokines [18]. In addition to EPA and DHA, potential bioactive novel fatty acids have been identified in blue mussel [17], as well as in green lipped mussel (*Perna Canaliculus*) [19]. In vitro studies have also shown anti-cyclooxygenase effects of lipids extracted from green lipped mussel or blue mussel [20]. In addition, not only the lipid fraction seems to have anti-inflammatory effects; water extracts from hard-shelled mussel and blue mussel, which contains among other substituents peptides and taurine, decreased inflammation in an in vivo model (zebrafish) [21], as well as an in vitro model, respectively [22]. These findings indicate that a combination of the lipids and the water fraction, i.e., the whole food, might be of interest as an anti-inflammatory food.

Overfishing is a worldwide global problem, and unfortunately, aquaculture depends on fish oil produced from fish. Currently, no means exist to produce long chain n-3 PUFA or fish containing these fatty acids in an economically- and environmentally-sustainable way. In contrast, blue mussel farming is beneficial for the environment since excessive nitrogen is removed at harvest and eutrophication of the sea is reduced.

Dietary interventions in general and in RA patients specifically face difficulties due to individual differences in habitual diet and differences in RA disease activity, diagnosis and co-morbidities. This makes a cross-over design optimal for such studies, since each individual is compared to him/herself, and inter-individual variation is thus minimized.

The aim of the randomized cross-over trial MIRA (Mussels, Inflammation and Rheumatoid Arthritis) was to test whether a diet rich in blue mussels, in addition to conventional medical treatment, could reduce disease activity in patients with established RA.

## 2. Materials and Methods

### 2.1. Subjects

**Figure 1:**
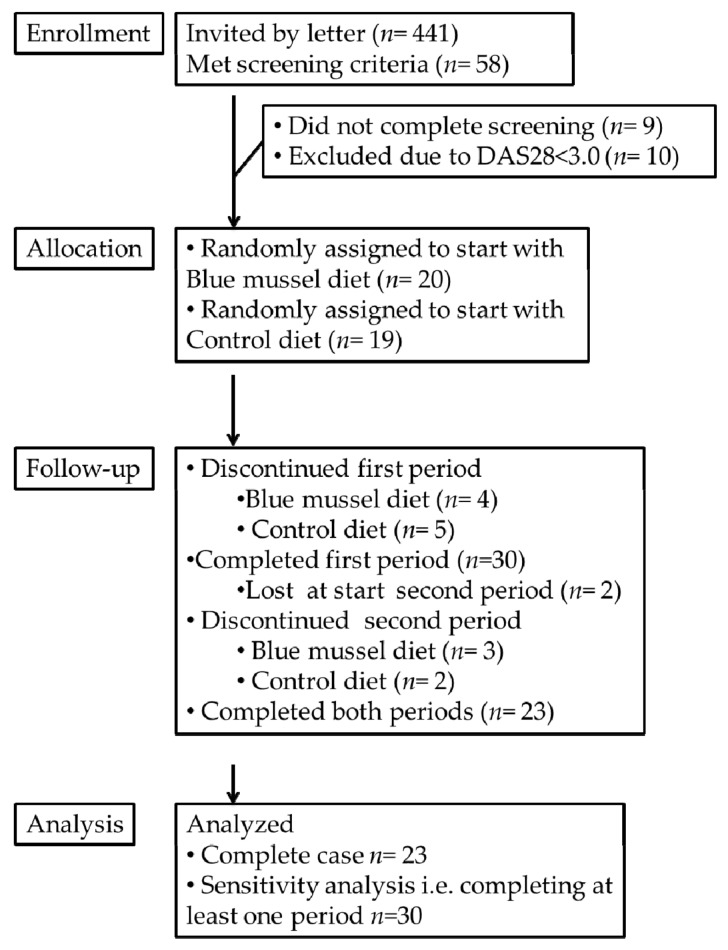
Consolidated standard reporting trial diagram of Mussels, Inflammation and Rheumatoid Arthritis (MIRA).

In summer 2015, 441 invitations were sent to women 25–65 years of age with established RA, i.e., disease duration >2 years, residing in the Västra Götaland Region, Sweden, and identified through the Swedish Rheumatology Quality Register (http://srq.nu). Among these, 58 women who responded to the invitation and met the inclusion criteria, willing to consume the study meals (including both blue mussels and meat) once a day, 5 days/week for 2 × 11 weeks, were invited for screening. At the screening visit, 39 women were considered eligible, i.e., on adequate medical treatment, body mass index (BMI) > 18 kg/m^2^ and moderate to high disease activity (disease activity score (DAS28) > 3.0) (Figure 1). All procedures were conducted according to the Declaration of Helsinki and approved by Gothenburg Regional Ethical Review Board (25 May 2015/Dnr 230-15), and all participants signed an informed consent.

The women were allocated by a computer-generated randomization list to start with either the blue mussel or control diet. Thirty women completed at least one period, and twenty-three women completed both dietary periods. Table 1 lists their baseline characteristics. Among these, 15 patients reported having reached menopause, and almost half the group was retired or unable to work due to their disease. Only two persons had parents born outside Europe. None smoked. Diagnosis and rheumatic drug treatment are presented in Table 2.

### 2.2. Experimental Design

The study was a randomized, single-blinded cross-over intervention with blue mussel meals or control meals for eleven weeks, respectively, and an eight-week washout period in between. Twenty vegetarian dishes plus 20 portions of either frozen blue mussels or meat (control) were delivered home every fourth week. Participants were instructed to consume one meal at lunch or dinner five days/week. It was not possible to blind study participants to type of intake, but personnel responsible for examinations, e.g., joint examinations and evaluation was blinded. Participants were instructed to follow their normal diet and to consume a maximum of two fish or shellfish meals a week besides the study meals. They were also instructed to be weight stable if possible. Compliance was evaluated by self-reporting number of dishes consumed and 24-h dietary recalls over the telephone midway each diet period. The first intervention period took place in fall 2015 and the second in spring 2016. Trial registration: clinical trials, https://register.clinicaltrials.gov, NCT02522052.

### 2.3. Dietary Intervention

To facilitate blue mussel consumption, 13 different nutritious accompanying dishes were developed. The dishes were specially produced at Gron Ko AB, Karlstad, Sweden, with the patented “MicVac method”, i.e., cooked, pasteurized, cooled and vacuum-packed [23]. Dishes were heated 2–4 min in a microwave before consumption. The thawed (or slightly warmed in the microwave) mussels or meat were mixed with the dish by the participants themselves. The dishes contained 1898 kJ (range 1652–2265 kJ) and a 75-g portion of blue mussels/meat 356/315 kJ. The mean ad libitum intake from the in total 119 delivered dishes (without meat or mussels) were 13.5 g protein, 20.4 g fat, 51.0 g carbohydrates, 5.5 g fiber, 10.7 g saturated fat, 6.4 g polyunsaturated fat and 1.6 g monounsaturated fat. Participants were told to consume the full mussel/meat portion.

The blue mussels (*Mytilus edulis*) were harvested in Limfjord, Denmark, pre-cooked, frozen and packaged by Vilsund Blue. To avoid seasonal differences, all mussel meat originated from the same batch and was kept at −18 °C at Gothenburg University from September 2015 until consumption. Pre-cooked salad chicken accompanied 80% of control meals and ham or beef meatballs the remaining 20%. These were chosen to provide as neutral an animal protein source as possible and to mimic blue mussel macronutrient composition, especially fat content. Each of the intervention meals provided about 21 E% proteins, 37 E% fat and 40 E% carbohydrates (about 2234 kJ/meal) with either blue mussels or meat.

Nutritional values of the meals were calculated using Dietist Net (Version 16.09.04, Kost och Näringsdata AB, Sweden) based on data from the Swedish National Food Administration and, for the blue mussels, from the Danish Food database (Fødevaredatabanken Version 2015.12.04, Technical University of Denmark). Calculated daily nutrient intakes from mussels and meat are shown in Table 3.

### 2.4. Clinical Response Variables and Participant Monitoring

As the primary outcome variable, the composite measure DAS28 calculated by using the formula: DAS28 = (0.56 × √(Tender joint count 28) + 0.28 × √(swollen joint count 28) + 0.70 × ln ESR) × 1.08 + 0.014 × VAS GH, which includes the number of swollen and tender joints out of 28, patients’ estimation of global health on a visual analog scale (VAS GH) and erythrocyte sedimentation rate (ESR) was chosen [24]. Joint examinations were performed by specially-trained research nurses at Clinical Rheumatology Research Centre, Sahlgrenska University Hospital, Gothenburg, Sweden. Response, i.e., treatment effect size was assessed by the EULAR (European League Against Rheumatism) response criteria [25,26]. Secondary outcome measures included DAS28-CRP (DAS28-CRP = (0.56 × √(Tender joint count 28) + 0.28 × √(swollen joint count 28) + 0.36 × ln (CRP+1)) × 1.08 + 0.014 × VAS GH + 0.96), pain and fatigue (VAS), disability (health assessment questionnaire (HAQ)) [27] and quality of life (SF-36) [28,29,30], which contains two main parts: mental component score (MCS) and physical component score (PCS). Furthermore, blood lipids, hemoglobin concentration, white blood cell and platelet counts were measured in fasting samples and immediately analyzed at Central Laboratory for Clinical Chemistry, Sahlgrenska University Hospital, Gothenburg, Sweden. Data were collected at all four time points, i.e., before and after each dietary period. At baseline, a socio-demographics, lifestyle and food frequency questionnaire (FFQ) and medication were included, as well as anthropometric measurements. All participants registered any changes or additions to their regular medication.

### 2.5. Statistical Methods

Statistical analyses were performed using SPSS Version 22 (SPSS Inc., Chicago, IL, USA). Comparisons of outcome variables between the two intervention diets (after each period) were made with paired Student’s *t*-test or with the Wilcoxon signed rank test when a non-normal distribution was expected. Data are presented as the median (first quartile, third quartile) with significance set at α = 0.05. For EULAR response criteria, Pearson’s chi-square test and Fisher’s exact test were used.

A sample size of 29 subjects was calculated to be sufficient to detect a 0.6 change in DAS28 with a power of 0.80 and α = 0.05.

A sensitivity analysis was performed on subjects completing at least one period (*n* = 30) for the main outcome and its components. For missing values at the second baseline (*n* = 2), values were carried forward from the first baseline. For missing outcome values (*n* = 7), the median change in the remaining subjects was added to baseline values for control diet, and the third quartile (i.e., mimicking a poor response as a conservative, worst case scenario) was added to baseline values for blue mussel diet. If the calculated values exceeded the biologically possible ranges, they were set to the possible limit (i.e., VAS > 100 mm was set to 100 mm and CRP < 0 was set to 0). Thereafter, DAS28 and DAS28-CRP were calculated from these new imputed values.

## 3. Results

### 3.1. Influence of Intervention on Disease Activity

**Figure 2:**
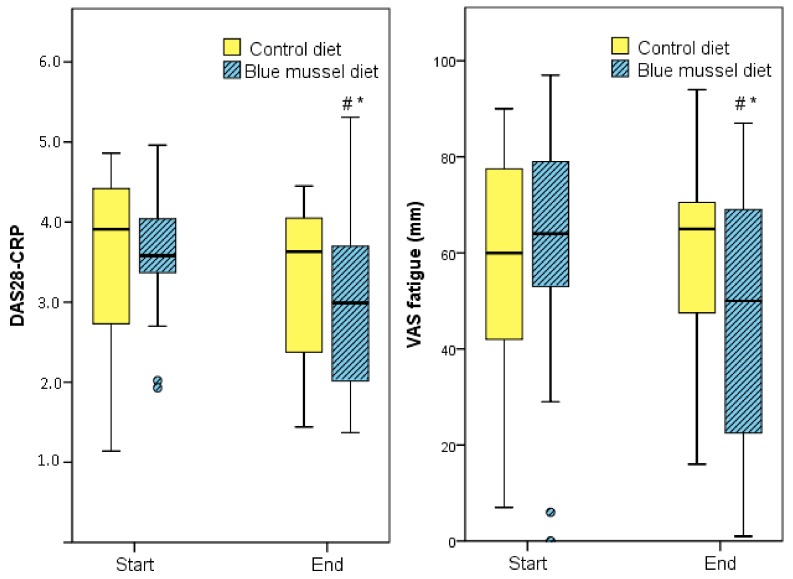
DAS28-CRP and visual analog scale (VAS) fatigue before and after intervention diets. DAS28-CRP. * Significant difference between the diets *p* = 0.048. # Significant difference between before and after the diet *p* = 0.007. VAS fatigue. * Significant difference between the diets *p* = 0.021. # Significant difference between before and after the diet *p* = 0.035.

**Figure 3:**
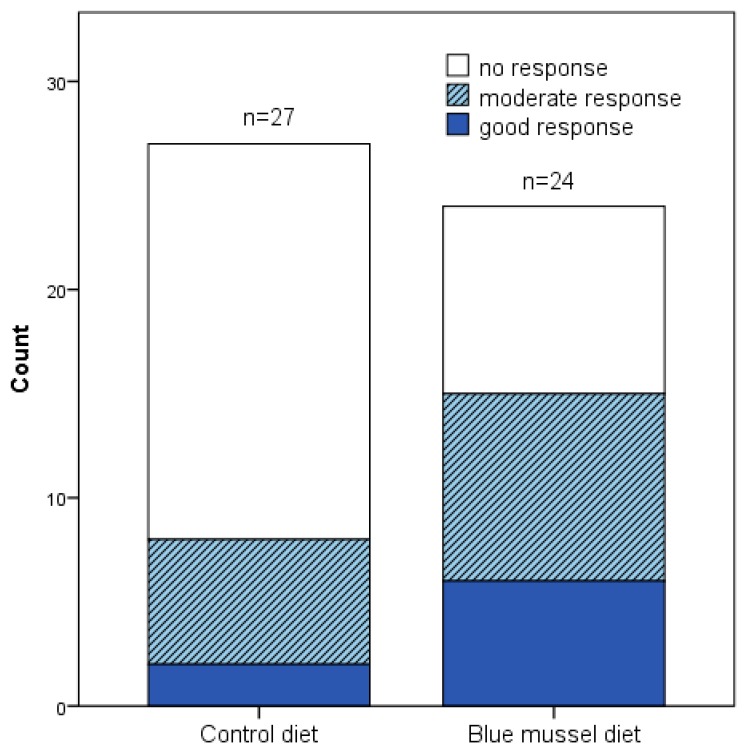
DAS28 EULAR-criteria response to blue mussel and control diet for all that completed at least one period. Pearson’s chi-square test for moderate, good or no response *p* = 0.049 and Fisher’s exact test *p* = 0.060 between the dietary periods. Control diet *n* = 27, blue mussel diet *n* = 24.

No significant difference was obtained between measures in DAS28 at the end of the two diet periods, respectively (*p* = 0.200; Table 4), although DAS28 decreased significantly during the blue mussel period (*p* = 0.017). Still, the blue mussel diet period was associated with a significantly lower DAS28-CRP than was the control diet period (*p* = 0.048), mainly due to fewer tender joints, lower CRP and a significant improvement in VAS global health during the mussel diet period; see Table 4 and Figure 2. In addition, there was a significant difference in EULAR response criteria (i.e., no, good or moderate response) between the diets (*n* = 27 control periods, *n* = 24 blue mussel periods, *n* = 30 individuals in total contributing to the periods; Pearson’s chi-square test *p* = 0.049 and Fisher’s exact test *p* = 0.060) in favor of blue mussel diet; Figure 3.

### 3.2. Influence of Intervention on Quality of Life and Disability

Quality of life measured as the SF-36 MCS summary score, including three out of four components (vitality, social functioning and mental health), as well as the general health component in the physical component score (PCS), showed significantly better health after the blue mussel diet compared to the control diet; Table 5. Bodily pain and role limitations due to physical health in PCS improved during the blue mussel period although not significantly compared to the control diet. In addition, VAS score for global health, pain and fatigue significantly improved after the blue mussel diet compared to the control diet; Table 5.

### 3.3. Influence of Intervention on Blood Lipids, Hemoglobin, White Blood Cell and Platelet Counts and Body Weight

There were no significant differences in levels of blood lipids (triacylglycerides, LDL, HDL, ApoA, ApoB), white blood cells, platelets or hemoglobin concentration at the end of the two dietary periods and also no significant changes during any of the dietary periods; Table 6. Body weight was maintained.

### 3.4. Habitual Dietary Intake of Participants

The control diet providing chicken for four meals a week and red meat once a week was in line with the participants’ reported habitual diet from the FFQ. The participants reported consuming fish and shellfish about twice a week before the study, with a range from less than once a month to six times a week. Blue mussels were normally consumed less than once a month.

### 3.5. Compliance, Drop-Out and Adverse Events

Reasons for drop-out included not being able or wanting to continue eating the dishes or blue mussels (*n* = 11) and sickness or absentee at visit (*n* = 5), Figure 1. Disease activity at baseline among participants who completed (*n* = 23) and drop-outs (*n* = 17) was similar, but VAS pain was higher among drop-outs. No differences in any outcome measures were detected between participants (*n* = 23) and the drop-outs who completed the first dietary period (*n* = 7). The 24-h recalls midway through each dietary period confirmed that the participants followed the dietary instructions. Self-report on weekly intake (number of meals/week (S.D.)) was 4.4 (0.38), indicating good compliance. There were no significant differences in changes in steroid dosage or DMARD treatments between the groups. Two cases of adverse events occurred in the form of teeth injury from biting in sand, small pieces of mussel shell or a pearl. No other adverse events or side effects were reported.

## 4. Discussion

This study is the first human intervention on blue mussel intake. The intention of this study was to evaluate possible positive effects on disease activity in women with RA from blue mussel intake. The results indicate that blue mussels, a foodstuff rich in selenium, iodine, B12 and containing marine n-3 PUFA, as well as other potential bioactive components, decrease disease activity, although not significantly, for DAS28, but for DAS28-CRP, in eleven weeks.

The sample size in the per protocol DAS28 paired analysis was few (*n* = 21), whereas a larger sample size was obtained in the sensitivity analysis (*n* = 30). The latter resulted in a significant result, indicating that the sample size of the per protocol analysis was too small. Indeed, the calculated required sample size was *n* = 29. DAS28 based on ESR was chosen as the primary outcome since this is a clinically-relevant outcome and it can also be used to estimate if the effect size is clinically significant based on the EULAR response criteria. Furthermore, when including all participants who completed at least one period, a statistically significant difference in numbers of EULAR no, moderate and good responders between the diets was found in favor of the blue mussel diet; Figure 3. This also strengthens the claim that the effect is clinically relevant.

The alternative outcome measure, DAS28-CRP, might underestimate disease activity [31], but even so, DAS28-CRP has some advantages over DAS28 based on ESR. Notably, CRP is not influenced by confounding factors such as age and fibrinogen levels to the same extent as is ESR and it reflects more short-term changes in disease activity [32]. The trend towards a reduction in CRP in this study (*p* = 0.106) is interesting, although the result must be confirmed by other inflammation markers. In a recent human intervention evaluating effects from lipid extract from hard-shelled mussel in patients with RA, no effects were seen on either ESR or CRP, but DAS28 (calculated without VAS GH), tumor necrosis factor α, interleukin 10 and prostaglandin E2 were improved after six months [18]. Marine n-3 PUFA is thought to decrease the production of inflammatory eicosanoids from arachidonic acid and promote the production of less inflammatory eicosanoids from EPA and other anti-inflammatory mediators such as resolvins [12]. Surprisingly, only leukotriene B4, among the inflammatory markers, was found to be significantly reduced in patients with RA by n-3 PUFA in a recent meta-analysis, but a possible explanation is the small number of studies including these markers [13]. It must be highlighted that the EPA and DHA intake in this study was about 0.3 g/day, which is considered a low dose, but effects could possibly be enhanced by other nutrients in the blue mussels such as selenium.

Further, the significant improvements in VAS global health, pain and fatigue, as well as the mental component of SF-36 including vitality, mental health and social functioning indicate that the reduction in disease activity might not be primarily due to a decrease in inflammation, but rather be mediated by other mechanisms. No solid evidence exists that marine n-3 PUFA have pain relieving effects in addition to anti-inflammatory effects, but it has been suggested that marine n-3 PUFA directly suppress pain, although the mechanisms are yet not fully understood [33,34,35]. Interestingly, an inverse relation between dietary n-3 fatty acid, i.e., fish intake, and resistant pain in patients treated with methotrexate was found in a recent Swedish cohort study [35]. In contrast, no association between n-3 PUFA supplementation and pain or anti-inflammatory effects was found. This is in line with our hypothesis that dietary n-3 PUFA intake (fish and shellfish) might be a better choice, since other nutrients such as antioxidants, peptides, vitamins and minerals will accompany the n-3 PUFA. These other nutrients, or a combination of them, could contribute to possible health effects, although there is little evidence to support this. For example; low selenium status has both been associated with high disease activity [36] and greater incidence of depression or negative mood [37]. Furthermore, there is mounting evidence that marine n-3 PUFA play a role in depression, but the scientific data from clinical trials remain inconclusive [38].

In the MIRA trial, there were no significant differences in weight, blood lipids or hemoglobin after either diet, which may be explained by the fact that the intervention meals replaced habitual meals that were similar in macronutrient content. This was confirmed with data from the FFQ. The participants’ habitual diets were healthier than are the habitual Swedish diets, as described in Riksmaten 2010, a population-based Swedish dietary survey [39]. Therefore, the control diet, although with a low amount of red meat, did not improve their diet. A low intake of red meat among women with RA has been shown in previous dietary questionnaire studies [40,41]. High doses of marine n-3 PUFA reduce triacylglycerides and improve lipoprotein profile [42], which also have been shown in patients with RA [43,44]. Such an effect from blue mussels, containing only a low dose n-3 PUFA, is thus not plausible.

### 4.1. Study Limitations

This study has several limitations. Blue mussels are, at least in Sweden, consumed regularly by few people, and we therefore suspected difficulties in recruiting participants and large study drop-out, which also was the case. The drop-outs tended to have a lower mentality score in SF-36, perhaps indicating less strength to commit and finish a study. The drop-outs who stayed in the study for the first period had similar disease improvements in this first period compared to the ones thereafter completing the full study, i.e., dropping out was not due to worsening of the disease.

There is risk for selection bias in this study in that the subjects recruited might only represent those strongly believing that diet alleviates their symptoms and that only subjects who enjoy eating blue mussels were selected. Still, the high drop-out indicates that the selection bias for enjoying blue mussels was not that high. Furthermore, individuals who strongly believe that diet influences their disease have most likely excluded certain foods or consumed a specific diet already before this study and were therefore not included, since all participants had to be omnivores and willing to consume all kinds of foods.

RA is characterized by flares, i.e., increased inflammatory activity. Furthermore, some pharmacological treatment, even if it is given regularly, has an increasing and decreasing effect over time. In addition, flares are often treated with steroids and/or a change of DMARDs. Thus, it is not possible to ascribe the effect in a study such as MIRA as entirely due to the intervention. Still, in order to evaluate whether beneficial effects were simply due to adjusted medication, this was thoroughly surveyed, and no significant differences in steroid dosage or DMARD treatments were found between the two diet periods.

It was not feasible to blind the participants to the intervention. Some of the significantly improved parameters (questionnaires and VAS) were collected through self-estimated scales. It is thus possible that these improvements represented placebo effects caused by patients knowing that they were on the active diet. Still, although not significant, the trend in CRP points towards a reduction in inflammation also when measured objectively, indicating that the results are not solemnly due to a placebo effect.

### 4.2. Study Strengths

This study is an original contribution to the field since no one has conducted a randomized controlled trial on the effect of blue mussel intake on RA before. The intervention was carefully performed, and the outcome measurements are clinically relevant. The two diets were matched on macronutrient composition and compliance evaluated. Study staff were blinded to diets. The participants had adequate pharmacological treatment before entering the study and are representative of women with RA in the source population. The cross-over design is the optimal choice for an efficacy dietary study, such as MIRA.

## 5. Conclusions

The MIRA study is the first human intervention on blue mussel intake. It suggests that an addition of blue mussels to the diet may complement the effect of pharmacological treatment on disease activity and reduce pain and fatigue in patients with RA. Further analysis of inflammation markers is needed to confirm possible anti-inflammatory effects. Furthermore, biomarkers in blood and urine need to be analyzed to identify potential bioactive compounds and hence explanations or mechanisms for the demonstrated improvement in disease activity and mental health. Lastly, all the results must be taken with caution since the intervention was not blinded to the participants, and the main outcome DAS28, although regarded as clinically relevant, includes subjective components.

## Figures and Tables

**Table 1:** Baseline characteristics. MCS, physical component score; PCS, physical component score.

Baseline Characteristics	Median (Q1, Q3) ^a^ *n* = 23	Range *n* = 23	Median (Q1, Q3) ^a^ *n* = 30	Range *n* = 30
Age (year)	55 (46, 63)	(32–66)	57 (51, 63)	(32–66)
Body mass index (kg/m^2^)	25.1 (23.2, 28.5)	(19.1–37.3)	25.4(23.1, 28.4)	(19.1–38.9)
Weight (kg)	68 (63, 83)	(51–90)	69 (62, 82)	(51–103)
Waist circumference	85 (80, 93)	(72–118)	84 (80, 93)	(72–118)
Hip circumference	101 (97, 110)	(85–122)	101 (97, 110)	(85–130)
Blood pressure systolic (mmHg)	125(120,140)	(100–160)	128(120,140)	(100–165)
Blood pressure diastolic (mmHg)	80 (80, 90)	(60–105)	80 (80, 90)	(60–105)
Serum cholesterol (mmol/L)	5.3 (4.7, 6.2)	(3.4–7.4)	5.4 (4.7, 6.3)	(3.4–8.0)
Serum low density lipoprotein (mmol/L)	3.2 (2.8, 4.1)	(2.2–4.9)	3.3 (2.7, 4.1)	(1.4–5.3)
Serum high density lipoprotein (mmol/L)	1.8 (1.4, 2.1)	(1.0–3.0)	1.9 (1.6, 2.3)	(1.0–3.0)
Serum triacylglycerides (mmol/L)	0.98 (0.61, 1.50)	(0.46–2.60)	0.94 (0.70, 1.58)	(0.46–2.60)
Apolipoprotein A1	1.7 (1.5, 2.0)	(1.2–2.4)	1.9(1.7, 2.0)	(1.2–2.4)
Apolipoprotein B	1.0 (0.8, 1.2)	(0.8–1.5)	1.0 (0.8, 1.2)	(0.8–1.2)
White blood cell count (×10^9^/L)	5.1 (4.3, 6.2)	(2.8–12.1)	5.7 (4.6, 7.2)	(2.8–12.8)
Platelet count (×10^9^/L)	269 (213, 322)	(168–559)	270 (214, 327)	(168–559)
Hemoglobin (g/L)	133(129, 140)	(116–145)	132(127, 139)	(114–151)
DAS28 ^b,c^	3.9 (3.5, 4.5)	(3.1–5.3)	4.0 (3.5, 4.6)	(3.1–6.1)
DAS28-CRP ^c^	3.6 (3.2, 4.2)	(2.0–4.8)	3.7 (3.2, 4.2)	(2.0–6.4)
Erythrocyte sedimentation rate (mm/1 h)	12.5 (5.75, 25)	(2–45)	14.0 (5.5, 23)	(2–45)
C-reactive protein (mg/L)	2 (1, 3)	(0–14)	2 (1, 3)	(0–39)
Swollen joints 28 (no)	2 (1, 2)	(0–7)	2 (1, 3)	(0–7)
Tender joints 28 (no)	4 (2, 8)	(0–21)	5 (2, 9)	(0–21)
VAS global health (mm) ^d^	54 (39, 66)	(22–78)	54 (41, 67)	(22–87)
VAS Pain (mm)	42(30, 64)	(7–82)	46(32, 64)	(7–93)
VAS Fatigue (mm)	64 (49, 75)	(16–90)	64 (48, 75)	(10–95)
Disability (HAQ) ^c,e^	1.13 (0.59, 1.25)	(0–2)	1.13 (0.62, 1.25) ^f^	(0–2)
SF-36 MCS (total score)	48 (39, 51)	(27–59)	48 (39, 52)	(27–62)
Vitality ^c^	25 (13, 44)	(6–56)	31 (13, 44)	(6–63)
Social functioning ^c^	63 (50, 75)	(12–100)	63 (50, 75)	(13–100)
Role limitations due to emotional problems ^c^	83 (50, 100)	(0–100)	79 (50, 100)	(0–100)
Mental health ^c^	60 (50, 70)	(35–85)	60 (53, 73)	(35–90)
SF-36 PCS (total score)	35 (30, 40)	(27–56)	35 (30, 41)	(25–56)
Physical functioning ^c^	45 (30, 65)	(25–100)	45 (29, 65)	(15–100)
Role limitations due to physical health ^c^	56 (31, 75)	(0–100)	56 (31, 70)	(0–100)
Bodily pain ^c^	41 (31,51)	(12–72)	41 (31,51)	(12–72)
General health ^c^	35 (30, 55)	(10–72)	35 (30, 55)	(10–72)

^a^ Q1 = first quartile, Q3 = third quartile of interquartile range (IQR); ^b^ *n* = 22; ^c^ score; ^d^ VAS = visual analogue scale; ^e^ HAQ = health assessment questionnaire; ^f^ *n* = 27.

**Table 2:** Diagnosis and rheumatic drug treatment.

Diagnosis:	*n* = 23	%	*n* = 30	%
Seropositive	13	57%	18	60%
Seronegative	10	43%	12	40%
**Rheumatic drug treatment:**				
Analgesic:	16	70%	20	67%
NSAIDs *n* (%)	15	65%	18	60%
DMARD (methotrexate), *n* (%)	14	61%	18	60%
Anti-TNF *n* (%)	5	22%	8	27%
Monoclonal antibody (MabThera) *n* (%)	4	17%	5	17%
Sulfasalazine: *n* (%)	4	17%	4	13%
Glucocorticosteroid *n* (%)	2	9%	6	29%

NSAIDs = Non-steroidal Anti-Inflammatory Drugs, DMARD = disease-modifying anti-rheumatic drugs, TNF = tumor necrosis factor.

**Table 3:** Calculated daily nutrient intake and percentage of recommended daily intake (RDI) from blue mussels or meat.

Nutrients Provided Daily:	Blue Mussels	Meat	Blue Mussels % RDI	Meat % RDI
Energy (kJ)	254	225	3%	3%
Energy (kcal)	61	54	3%	3%
Protein (g)	9.5	11	12%	14%
Fat (g)	1.5	1.2	2%	2%
Carbohydrates (g)	2.2	0.1	1%	0%
Saturated fatty acids (g)	0.24	0.40	1%	2%
Mono unsaturated fatty acids (g)	0.14	0.50	0%	2%
Poly unsaturated fatty acids (g)	0.47	0.22	3%	1%
EPA (fatty acid 20:5) (g)	0.18	0.00		
DHA (fatty acid 22:6) (g)	0.16	0.01		
Iron (mg)	2.64	0.17	18%	1%
Calcium (mg)	25.6	3.39	3%	0%
Zinc (mg)	1.54	0.50	22%	7%
Selenium (µg)	33.2	5.8	66%	12%
Iodine (µg)	117	1.34	78%	1%
Retinol EQ	33.5	4.99	5%	1%
Vitamin D (µg)	0.01	0.24	0%	2%
Vitamin B12 (µg)	12.1	0.13	603%	6%

Eicosapentaenoic acid (EPA), docosahexaenoic acid (DHA); data from the Danish Food database. Fødevaredatabanken Version 4, December 2015. Technical University of Denmark. Recommended daily intake (RDI) based on the Nordic Nutrition Recommendations 2012.

**Table 4:** Blue mussel diet versus control diet on disease activity.

	Control Diet			Blue Mussel Diet			
	Before ^a^	After ^a^	Δ ^a^	Before ^a^	After ^a^	Δ ^a^	*p* ^b^
DAS28 ^c,d^	3.81 (3.16, 3.73)	3.77 (2.69, 4.22)	−0.15 (−0.72, 0.21)	3.75 (3.15, 4.53)	3.40 (2.41, 3.73)	−0.72 (−1.5, −0.06) ^f^	0.200
SA	3.95 (3.35, 4.53)	3.88 (2.83, 4.23)	−0.16 (−0.74, 0.22)	3.96 (3.14, 4.53)	3.40 (2.52, 3.76)	−0.81 (−1.5, −0.08) ^e^	0.023 *
DAS28-CRP ^d^	3.91 (2.50, 4.54)	3.63 (2.36, 4.21)	−0.19 (−0.83, 0.17)	3.58 (3.35, 4.06)	2.99 (1.88, 3.72)	−0.50 (−1.69, −0.02)	0.048 *
SA	3.92 (2.86, 4.56)	3.5 (2.7, 4.2)	−0.18 (−0.81, 0.11)	3.61 (3.35, 4.16)	3.0 (2.2, 3.7)	−0.50 (−1.42, −0.01)	0.008 *
ESR (mm/1 h)	12.0 (5.0, 25.0)	8.0 (6.0, 25.0)	0.0 (−3.0, 2.0)	12.5 (5.0, 25.5) ^c^	9.0 (4.8, 19.3) ^c^	−1.0 (−5.5, 1.0) ^e^	0.952
SA	14.0 (5.0, 25.0)	8.0 (6.8, 25.0)	0.0 (−3.0, 2.0)	13.0 (5.0, 25.0)	9.0 (5.0, 19.3)	−0.5 (−5.3, 1.0)	0.289
CRP (mg/L)	2.0 (0.0, 3.0)	2.0 (0.0, 3.0)	0.0 (−1.0, 1.0)	2.0 (1.0, 5.0)	1.0 (1.0, 3.0)	−1.0 (−1.0, 1.0)	0.106
SA	2.0 (0.0, 4.8)	2.0 (0.8, 4.3)	−0.2 (−0.8, 0.11)	2.5 (1.0, 5.3)	2.0 (0.8, 3.0)	−1.0 (−1.5, 1.0)	0.080
SJ 28 (no)	2 (1, 3)	2 (0, 4)	0 (−2, 1)	2 (1, 3)	1 (0, 2)	−1 (−3, 1)	0.363
SA	2 (1, 3)	1.5 (0, 4)	0 (−2, 1)	2 (1, 3)	1 (0, 2.2)	0 (−3, 1)	0.192
TJ 28 (no)	3 (1, 12)	4 (0, 7)	−1 (−4, 1)	4 (2, 7)	2 (1, 5)	−2 (−5, 0)	0.075
SA	3.5 (1, 12)	4 (0.8, 6.3)	−1 (−4, 1)	4.5 (2, 8)	3 (0.8, 5.0)	−1 (−5, 0)	0.087
VAS GH (mm)	51 (31, 65)	40 (22, 57)	−2 (−15, 18)	57 (37, 69)	30 (11, 58)	−11 (−35, 5)	0.041 *
SA	51 (31, 65)	52 (34, 67)	1 (−9, 19)	56 (41, 69)	39 (14, 60)	−9 (−33, 5)	0.005 *
VAS pain (mm)	25 (15, 54)	46 (19, 68)	−5 (−12, 8)	51 (30, 72)	25 (15, 54)	−15 (−25, 3)	0.048 *
SA	47 (32, 65)	54 (25, 71)	−2 (−10, 9)	51 (32, 72)	37 (15, 54)	−12 (−25, 3)	0.004 *
VAS fatigue (mm)	60 (39, 78)	65 (47, 72)	−12 (−18, 19)	64 (49, 82)	50 (20, 69)	−12 (−37, 10)	0.021 *
SA	58 (38, 77)	65 (48, 73)	−1 (−14, 19)	63 (49, 78)	52 (28, 71)	−5 (−25, 9)	0.084

SA = sensitivity analysis (*n* = 30); ESR = erythrocyte sedimentation rate; CRP = C-reactive protein, SJ = swollen joints; TJ = tender joints; VAS = visual analogue scale; GH = global health; *n* = 23; ^a^ median and interquartile range (first quartile, third quartile); ^b^ *p*-values are calculated with the Wilcoxon signed rank test between after control and after blue mussel diet; * *p* < 0.05; **p < 0.02; ^c^ *n* = 22; ^d^ score; ^e^ *n* = 21.

**Table 5:** Blue mussel diet versus control diet on quality of life.

	Control Diet			Blue Mussel Diet			
	Before ^a^	After ^a^	Δ ^a^	Before ^a^	After ^a^	Δ ^a^	*p* ^b^
SF-36 MCS (total score)	48 (41, 51)	46 (41, 54)	0 (−6, 7)	43 (38, 52)	50 (47, 56)	6 (2, 13)	0.005 **
Vitality ^c^	31 (19, 44)	38 (19, 50)	6 (−13, 19)	31 (13, 44)	50 (31, 63)	13 (4, 25)	0.012 *
Social functioning ^c^	63 (50, 88)	63 (50, 88)	0 (−13, 13)	63 (38, 75)	75 (50, 100)	13 (0, 38)	0.04 *
RL emotional problems ^c^	75 (50, 100)	75 (50, 100)	0 (−17, 17)	75 (50, 100)	79 (75, 100)	8 (0, 27)	0.145
Mental health ^c^	60 (55, 75)	65 (55, 80)	0 (−5, 15)	65 (50, 70)	70 (60, 85)	10 (0, 20)	0.008 **
SF-36 PCS (total score)	38 (33, 44)	37 (33, 44)	0 (−2, 4)	35 (27, 41)	38 (32, 48)	3 (−3, 7)	0.503
Physical functioning ^c^	50 (35, 74)	50 (35, 65)	0 (−5, 5)	45 (30,65)	50 (35,75)	5 (−5, 15)	0.299
RL physical health ^c^	63 (44, 81)	50 (50, 88)	6 (−13, 13)	44 (31, 75)	69 (50, 88)	13 (−6, 27)	0.442
Bodily pain ^c^	41 (22, 62)	51 (31, 62)	15 (−9, 28)	41 (31, 51)	42 (41, 62)	10 (0, 21)	0.599
General health ^c^	35 (30, 57)	35 (25, 52)	−5 (−10, 10)	35 (30, 55)	47 (25, 67)	3 (−5, 17)	0.03 *
HAQ ^d^	1.13 (0.44,1.28)	1.00 (0.50,1.38)	0 (−0.25,0.16)	1.06 (0.47,1.25)	1.00 (0.13,1.28)	−0.13 (−0.38,0.00)	0.088

RL = role limitations due to; *n* = 23; ^a^ median and interquartile range (first quartile, third quartile); ^b^ *p*-values are calculated with the Wilcoxon signed rank test between after control and after blue mussel diet. * *p* < 0.05. ** *p* < 0.02. ^c^ score.

**Table 6:** Blue mussel diet versus control diet in blood lipids, blood count, hemoglobin and BMI.

	Control Diet			Blue Mussel Diet			
	Before ^a^	After ^a^	Δ ^a^	Before ^a^	After ^a^	Δ ^a^	*p* ^b^
BMI (kg/m^2^)	25.6 (23.6,27.8)	25.1 (23.6,28.1)	0.00 (−0.35,0.35)	24.8 (22.9,28.5)	25.1 (23.1,28.3)	0.08 (−0.38,0.40)	0.852
Cholesterol ^c^	5.4 (4.8, 6.2)	5.4 (4.5, 6.3)	−0.2 (−0.4, 0.2)	5.3 (4.6, 6.2)	5.2 (4.8, 6.2)	0.1 (−0.3, 0.3)	0.131
LDL^c^	3.3 (3.0, 4.1)	3.3 (2.5, 4.1)	−0.1 (−0.2, 0.1)	3.1 (2.6, 4.1)	3.2 (2.7, 4.2)	0.0 (−0.2, 0.3)	0.359
HDL^c^	1.8 (1.4, 2.2)	1.7 (1.4, 2.4)	−0.1 (−0.2, 0.1)	1.9 (1.3, 2.2)	1.8 (1.4, 2.2)	0.0 (−0.2, 0.1)	0.336
TAG ^c^	1.00 (0.72, 1.50)	0.85 (0.67, 1.40)	−0.09 (−0.21,0.10)	1.00 (0.71, 1.40)	0.99 (0.71, 1.50)	−0.02 (−0.20,0.11)	0.263
Apo A1 ^c^	1.7 (1.6, 2.0)	1.8 (1.4, 2.0)	0.0 (−0.2, 0.1)	1.7 (1.4, 2.1)	1.8 (1.5, 2.0)	0.0 (−0.1, 0.1)	0.486
Apo B^c^	0.99 (0.85, 1.20)	0.96 (0.81, 1.20)	0.0 (−0.1, 0.1)	1.00 (0.82, 1.20)	0.92 (0.83, 1.30)	0.0 (−0.1, 0.1)	0.432
WBC (*10^9^/L)	5.0 (4.4, 6.0)	6.0 (4.6, 6.6)	0.3 (−0.5, 0.0)	5.3 (4.1, 6.2)	5.1 (4.6, 6.2)	0.2 (−0.2, 0.5)	0.076
PC (*10^9^/L)	271 (224, 317)	277 (229, 309)	5 (−18, 28)	269 (236, 322)	282 (228, 331)	10 (−20, 27)	0.513
Hb (g/L)	136 (128, 143)	137 (127, 143)	0 (−3, 5)	131 (129, 140)	135 (125, 140)	1 (−6, 6)	0.088

Δ = difference; BMI = body mass index; LDL = serum low density lipoprotein; HDL = serum high density lipoprotein; TAG = serum triacylglycerides; Apo = apolipoprotein; WBC = white blood cell count; PC = platelet count; Hb = hemoglobin; *n* = 23; ^a^ median and interquartile range (first quartile, third quartile); ^b^ *p*-values are calculated with the Wilcoxon signed rank test between after control and after blue mussel diet; ^c^ (mmol/L).
